# Effects of Nitrogen Application in the Wheat Booting Stage on Glutenin Polymerization and Structural–Thermal Properties of Gluten with Variations in HMW-GS at the *Glu-D1* Locus

**DOI:** 10.3390/foods9030353

**Published:** 2020-03-18

**Authors:** Lijun Song, Liqun Li, Liye Zhao, Zhenzhen Liu, Xuejun Li

**Affiliations:** State Key Laboratory of Crop Stress Biology in Arid Areas and College of Agronomy, Northwest A&F University, Yangling 712100, Shaanxi, China; lijunsongNWAFU@163.com (L.S.); liliqun@nwsuaf.edu.cn (L.L.); liyezhao5526@163.com (L.Z.); zhenzhenliu1717@163.com (Z.L.)

**Keywords:** wheat booting stage, gluten structural‒thermal characteristics, glutenin polymerization, high-molecular-weight glutenin subunits, nitrogen application

## Abstract

Wheat gluten properties can be improved by the application of nitrogen. This study investigates the effects of nitrogen application in the booting stage on glutenin polymerization during grain-filling and structural–thermal properties of gluten based on the high-molecular-weight glutenin subunits (HMW-GSs) using near-isogenic lines (*Glu-1Da* and *Glu-1Dd*). The nitrogen rate experiment included rates of 0, 60, 90, and 120 kg N ha^−1^ applied with three replicates. Nitrogen significantly improved the grain quality traits (wet gluten contents, Zeleny sedimentation values, and maximum resistance) and dough strength (dough development time, dough stability time, and protein weakening), especially in wheat with the *Glu-1Da* allele. Nitrogen increased the protein composition contents, proportions of glutenins and HMW-GSs, and disulfide bond concentration in the flours of *Glu-1Da* and *Glu-1Dd*, and accelerated the polymerization of glutenins (appearing as glutenin macropolymer) during grain-filling, where nitrogen enhanced the accumulation and polymerization of glutenins more for line containing *Glu-1Da* than *Glu-1Dd*. The β-sheets, α-helix/β-sheet ratio, microstructures, and thermal stability were also improved to a greater degree by nitrogen for gluten with *Glu-1Da* compared to *Glu-1Dd*. Nitrogen treatment was highly effective at improving the gluten structural‒thermal properties of wheat in the booting stage, especially with inferior glutenin subunits.

## 1. Introduction

Bread wheat (*Triticum aestivum* L.) is the most widely grown cereal crop throughout the world, where it provides 20% of the total calories and 22% of the total protein, on average, in the human diet [1]. Wheat flour has extensive uses in food products, mainly because of the unique properties of gluten, which is divided into glutenins and gliadins, where the glutenins contribute mainly to the elasticity of wheat dough and the gliadins to the viscosity [2]. Due to gradual improvements in the quality of life, the requirements for a higher quality of wheat flour have grown to ensure increased acceptance by consumers. Although wheat breeders attempt to select high quality varieties with strong gluten and high yield, many low-quality wheat varieties still persist. Therefore, numerous studies have focused on improving the grain quality in wheat production [3,4]. The quality of wheat is affected by the genotype, growing conditions, and their interactions [5]. In particular, fertilization can be regulated more easily to affect the quality in wheat production, especially fertilization with nitrogen (N).

The grain protein content (GPC) (especially storage proteins) of wheat is the main determinant of the end-use quality and it is controlled by both genetic and environmental factors. Since the availability of N has a greater effect on the GPC than genetic factors [6], increasing the total dose of N is an effective practice for increasing the protein content and its fractions in wheat. However, excessive N application reduces N use efficiency and increases the risk of environmental pollution [7], supplementary agronomic methods should be explored to reduce these concerns. Previous studies have applied various nitrogen fertilizer strategies in terms of the rates, timing, type of N fertilizer, and soil N availability [4,8] to better utilize the applied N and increase the GPC in wheat production. The split of N fertilizer and the modification of topdressing time increases the GPC and gluten proportions without increasing the N dose [6,7]. The application of N fertilizer in the jointing stage significantly increases the ratios of glutenin relative to gliadin (glu/gli) and high molecular weight glutenin subunits (HMW-GS) to low molecular weight glutenin subunits (LMW-GS) (HMW/LMW-GS) [9,10], and improves the end-use quality of wheat [8]. Moreover, the booting stage is the key period of plant vegetative growth to reproductive growth, where the flag leaf of wheat reaches the final leaf size and the highest photosynthetic capacity [11]. Recent study suggests that N topdressing in the top-first-leaf stage greatly improves the grain yield and gluten protein content compared to other development stages of wheat [4].

The proportion of the largest glutenin polymers, known as the sodium dodecyl sulfate (SDS)-unextractable polymeric proteins (UPP) or glutenin macropolymer (GMP), is used as an indicator of the glutenin polymer size distribution and is positively associated with the strength and elasticity of dough [12]. The formation of glutenin polymers is mainly controlled by the genotype during wheat grain development. A previous study used near isogenic lines (NILs) to investigate the effects of HMW-GS on glutenin polymerization and found that the strength-associated subunits (Dx5 + Dy10) resulted in earlier polymerization and a higher UPP content compared with its counterparts (Dx2 + Dy12) [13]. Moreover, it is also affected by different environmental factors (especially N fertilizer). The optimum amount of N application accelerates the polymerization of glutenin during grain filling in the sowing or jointing stage, which differs between varieties [10,14,15]. However, few studies have investigated the effect of N fertilizer application on the polymerization of glutenin during wheat grain development in the booting stage, especially in terms of the strong/weak-associated subunits with an identical genetic background.

The structural and thermal properties of gluten proteins have crucial effects on the baking quality of dough. Studies have investigated the effects of various treatments on the secondary and tertiary structures of gluten to improve the processing quality of wheat dough. Pulsed light promotes the partial de-polymerization of hydrated gluten proteins via disulfide bond (-S-S-) exchange to affect the functionality of gluten [16]. Moderate temperatures induce changes in the secondary structure of matured gluten proteins conditioned at different water contents [17]. Adding polysaccharides affects the secondary structure of gluten via the interaction between polysaccharide and gluten molecules, as well as affects the thermal properties of gluten proteins [18]. However, the effects of N fertilizer application on the structural and thermal characteristics of wheat gluten are unclear, especially application of N in the booting stage

The aims of this study are to investigate the effect of N fertilizer in the booting stage on the quality formation of wheat with HMW-GS variation at the *Glu-D1* locus by determining the glutenin polymerization during grain development and gluten structural–thermal properties; to compare the difference between the wheat with *Glu-1Da* (inferior glutenin subunits) and *Glu-1Dd* (superior glutenin subunits) in response to N fertilizer applied in the booting stage, thereby providing a reference for the high quality cultivation of wheat, especially with inferior glutenin subunits.

## 2. Materials and Methods

### 2.1. Plant Materials

NILs were created by initially crossing Xinong 1718 (Ax1, Bx7 + By9, Dx2 + Dy12) with wheat cultivar Fa 710 (Ax2*, Bx6 + By8, Dx5 + Dy10) and Fa 790 (Ax1, Bx6+By8, Dx5+Dy10), respectively. The F2 progenies were analyzed by sodium dodecyl sulfate polyacrylamide gel electrophoresis (SDS–PAGE) to select the various HMW-GS compositions and then backcrossed with Xinong 1718 six times. The backcross F1 progenies with the appropriate HMW-GS were selected. BC_6_F_11_ homozygous NILs were checked for glutenin and gliadin by SDS–PAGE and acid‒PAGE (A‒PAGE). The two NILs (*Glu-1Da* and *Glu-1Dd*) were selected for this study, which exhibits similar agronomic traits. The wheat varieties Pompei (null, Bx6 + By8, Dx2 + Dy12), Jin47 (null, Bx7 + By9, Dx2 + Dy12), Guadalupe (null, Bx13 + By19, Dx5 + Dy10), and Lankao Teaizao (Ax1, Bx7 + By9, Dx2 + Dy12) were selected as controls for identifying the HMW-GS compositions.

### 2.2. Experimental Design

Field experiments were conducted at Yangling (108°40′E, 34°160′N), Shaanxi Province, China, during growing seasons in 2015–2016 and 2016–2017. The *Glu-1Da* and *Glu-1Dd* were manually planted individually in the medium fertile soil (nitrogen content of 0.8095‰) with a sowing density of ninety seeds per row. The block size was 10 × (3 m × 0.23 m) and row space of 0.23 m. A randomized complete block design with three replicates and four different N management strategy treatments was employed. The treatments were designed as the 0, 60, 90, and 120 kg N ha^−1^ in form of urea applied in the booting stage. The basic fertilizer applied before sowing comprised 360 kg chelated organic fertilizer (NY525-2012, N + P_2_O_5_ + K_2_O ≥ 5%, amino acids ≥ 10%, organic matter ≥ 45%, Xianghui Agricultural Technology Development Co. Ltd., Hunan, China) per ha and 685 kg diammonium phosphate (HG/T4132-2010, 21.2% N and 53.8% P_2_O_5_, Shifang Kanglong Chemical Co. Ltd., Sichuan, China) per ha.

Spikes obtained from *Glu-1Dd* and *Glu-1Da* were immediately tagged when the upper part of the floret spikelet reached anthesis in the morning. About 30 labeled spikes were randomly collected at 4, 7, 10, 16, 22, 28, and 34 days after anthesis (DAA). Seeds were stripped immediately from the central part of the spikes and ground into a fine powder after lyophilization. The powder was stored at −40 °C until further analysis. The harvested mature grains were sun dried and stored for 60 days, milled using a multifunctional pulverizer (Y400, Laobenxing, Zhejiang, China), and then screened through a 100-mesh sieve.

### 2.3. Separation and Identification of Gliadin and Glutenin

Glutenins and gliadins were extracted and separated from the milled flour produced from *Glu-1Dd* and *Glu-1Da* using previous methods [19,20]. The glutenins from the two NILs were further separated and identified by reversed phase high performance liquid chromatography (RP-HPLC) analysis using the method described by Liu et al. [20].

### 2.4. Near-infrared Reflectance (NIR) Analysis of Grains

A NIR instrument (Diode array 7200, Perten Instruments AB, Sweden) was used to determine the wet gluten content, Zeleny sedimentation value, and maximum resistance for the mature grains from wheat containing *Glu-1Dd* and *Glu-1Da*. Each sample was tested in triplicate.

### 2.5. Formation of Total Protein and Its Compositions during Grain Development

The contents of total protein in the *Glu-1Dd* and *Glu-1Da* grains at different stages (4, 7, 10, 16, 22, 28, and 34 DAA and maturity) were measured using the Kjeldahl method. The gliadin and glutenin in grains at the eight stages were sequentially extracted according to Song et al. [21], the contents were measured using the Kjeldahl method. The GMP contents of grains in eight different stages were measured using a previously reported method [10]. The ratio of HMW-GS to LMW-GS in matured grains was determined according to our previous report [20]. Each sample was tested in triplicate.

### 2.6. Determination of Nitrogen Use Efficiency (NUE) and Grain Yield

The nitrogen contents of above-ground part at anthesis and harvest were determined using the Kjeldahl method. The grain yield (GY) for each block was weighted accurately, and then converted into the yield per ha. Plant N uptake (PNU) was calculated as the difference between nitrogen content in above-ground part at anthesis and harvest. The recovery efficiency (RE), NUE–grain yield, NUE–total protein yield, and NUE–GMP yield were calculated using equations:

RE (%) = (PNU_N_ − PNU_0_)/N_fer_,

NUE–grain yield (kg/kg) = (GY_N_ − GY_0_)/N_fer_,

NUE–total protein yield (kg/kg) = (TPY_N_ − TPY_0_)/N_fer_,

NUE–GMP yield (kg/kg) = (GMPY_N_ − GMPY_0_)/N_fer_,

where PNU_N_ and PNU_0_ are PNU in N-fertilizer and non-fertilizer, respectively; GY_N_ and GY_0_ are grain yield (GY) in N-fertilizer and non-fertilizer, respectively; TPY_N_ and TPY_0_ are grain total protein yield (TPY, calculated as GY multiplied by total protein content) in N-fertilizer and non-fertilizer, respectively; GMP_N_ and GMP_0_ are GMP yield (GMPY, calculated as GY multiplied by GMP) in N-fertilizer and non-fertilizer, respectively; and N_fer_ is N from the applied fertilizer. Each sample was tested in triplicate.

### 2.7. Preparation of Gluten Samples

Gluten was obtained from matured ground wheat flour according to the previous methods [20] with minor modifications. Briefly, flour (50 g) was mixed with an appropriate amount of water (flour (g): water (mL) = 3:2−5:2) for 20 min to form even dough. The dough was washed with distilled water until no starch remained. The gluten obtained was freeze dried and then milled to a fine powder with a pestle and mortar. The fine gluten powder was stored in a refrigerator at −40 °C until further analysis.

### 2.8. Determination of Sulfhydryl Groups (-SH) and Disulfide Bond (-S-S-)

The -SH and -S-S- were determined according to the method [22] with some modifications. Freeze‒dried gluten (30 mg) was dispersed in 8 mL Tris–Gly buffer (pH 8.0, consisting of 86 mM Tris, 92 mM Gly, 8 M urea, 4 mM EDTA, and 1% SDS), agitated and extracted for 1 h at room temperature. The suspension was centrifuged at 13,600 g for 10 min to remove the particulate and the supernatant was collected for the determination of -SH and -S-S-. The following procedure is consistent with Guo et al. [22]. Each sample was tested in triplicate.

### 2.9. Fourier Transform Infrared Spectroscopy (FTIR) Analysis of Gluten

FTIR spectroscopy was employed to determine the secondary structures of the freeze-dried gluten samples. Infrared spectra were recorded at 25 °C between 1700 and 1600 cm^−1^ using a FTIR spectrometer (Vertex 70, Bruker, Germany) with a spectral resolution of 4 cm^−1^ as described by Liu et al. [20]. Dried flour powder (1 mg) and finely ground potassium bromide (100 mg) (Sigma-Aldrich, Milan, Italy) were weighed accurately and homogenized using an agate mortar and pestle. Approximately 20 mg of the mixture was pressed into thin sheets with a 10 ton hydraulic press. The main structures and spectral band ranges were fitted as described previously [17,23], where they comprised: intermolecular β-sheets at 1612–1620 cm^−1^; β-sheets at 1625–1642 cm^−1^; α-helices at 1650–1660 cm^−1^; β-turns at 1660–1670 cm^−1^; and antiparallel β-sheets at 1675–1695 cm^−1^. Each sample was tested in triplicate.

### 2.10. Thermal Properties of Gluten

The thermal properties of the gluten were conducted with a thermal analysis instrument (STA7200RV, HITACHI, Tokyo, Japan) according to previously described methods [18]. Freeze-dried gluten samples (7–9 mg) were weighed in an aluminum pan and heated from 25 °C to 600 °C at a heating rate of 20 °C min^−1^ under nitrogen gas at a flow rate of 80 mL min^−1^. The denaturation peak temperatures (T_p_), enthalpy of thermal transition (ΔH), the degradation temperature (T_d_), and weight loss were determined using the TA7000 software (version 10.41, Hitachi, Tokyo, Japan). Each sample was tested in triplicate.

### 2.11. Dough Mixing Properties

A Mixolab system (Chopin, Tripette and Renaud, France) was used to analyze the mixing properties of dough obtained from matured wheat. Measurements were performed using the Mixolab “Chopin +” protocol described by Dhaka et al. [24]. The parameters recorded by the Mixolab system comprised: (1) dough development time (min), (2) dough stability time (min), (3) protein weakening based on mechanical mixing and temperature increase, i.e., decrease in dough consistency due to excessive mixing, (Nm). Each sample was tested in triplicate.

### 2.12. Microstructure Analysis of Wheat Dough Using Confocal Laser Scanning Microscopy (CLSM) and Image Analysis

To quantify the microstructure and three-dimensional structure of the gluten protein network, freshly prepared dough samples was observed by CLSM according to a method established in our laboratory [25]. Briefly, 10 g of flour were mixed with 4 mL water and 1 mL of Rhodamine B solution (0.01 g/100 mL water) for 10 min. The dough samples were transferred to an object carrier and sealed with a cover glass. Samples were analyzed by CLSM system (Olympus, Tokyo, Japan). Ten CLSM images were analyzed using AngioTool64 version 0.6a (National Cancer Institute, National Institute of Health, Maryland, USA) according to the method described by Gao et al. [25]. The gluten microstructure was quantified using several parameters, including protein area, protein junctions, junction density, total protein length, protein end-points, lacunarity, branching rate, and endpoint rate.

### 2.13. Statistical Analysis

Statistical analyses of the results were conducted using SPSS 22.0 (SPSS Inc., Chicago, IL, USA). All of the data were subjected to single-factor analysis of variance and significant differences (*p* < 0.05) among the parameters considered were tested using Duncan’s multiple range tests. Figures were generated using Sigmaplot 12.5 (Systat Software Inc., San Jose, CA, USA) and Adobe Photoshop 7.0 (Adobe Systems Inc., San Jose, CA, USA). All the data are presented as the mean of two years.

## 3. Results and Discussion

### 3.1. Identification of HMW-GS Compositions

The gliadin and glutenin compositions were separated and confirmed for the lines with *Glu-1Dd* and *Glu-1Da* by A-PAGE and SDS-PAGE, respectively (Supplementary Figure S1). The HMW-GS compositions of *Glu-1Dd* (Dx5 + Dy10) and *Glu-1Da* (Dx2 + Dy12) only differed at the *Glu-D1* locus and were consistent with Ax1 at *Glu-A1* and Bx6 + By8 at *Glu-B1*, whereas the LMW-GS and gliadin compositions were identical (Figure S1A,B). To further verify the difference in glutenin only at HMW-GS, the glutenin compositions were analyzed by RP-HPLC. The elution profiles for the glutenin from *Glu-1Dd* and *Glu-1Da* were identical except for the HMW-GS region (Figure S1C), thereby agreeing with the results obtained by SDS-PAGE. Therefore, the two wheat lines used in this study were NILs with only variations in HMW-GS at the *Glu-D1* locus.

### 3.2. Analysis of Nitrogen Use Efficiency and Grain Yield of Wheat Lines

The plant nitrogen uptake (PNU), recovery efficiency (RE), grain yield, nitrogen use efficiency (NUE)–grain yield, NUE–total protein yield, and NUE–GMP yield under different nitrogen strategies to analyze nitrogen absorption of *Glu-1Da* and *Glu-1Dd* (Supplementary Figure S2). The nitrogen application increased significantly the PNU, RE, NUE‒total protein yield, NUE‒GMP yield, grain yield and, NUE–grain yield of lines containing *Glu-1Dd* and *Glu-1Da*, except for NUE‒GMP yield for *Glu-1Dd* at 120 kg N ha^−1^, and had no significant effects on the agronomic traits (e.g., plant height, leaf type) (data not shown). These results suggested that nitrogen application at booting stage used in our experiment increased significantly the nitrogen absorption and conversion rate of wheat and had no obvious damage to wheat growth.

### 3.3. Grain Quality Traits of Wheat with Variations in HMW-GS at the Glu-D1 Locus Under Different N Treatments

**Figure 1:**
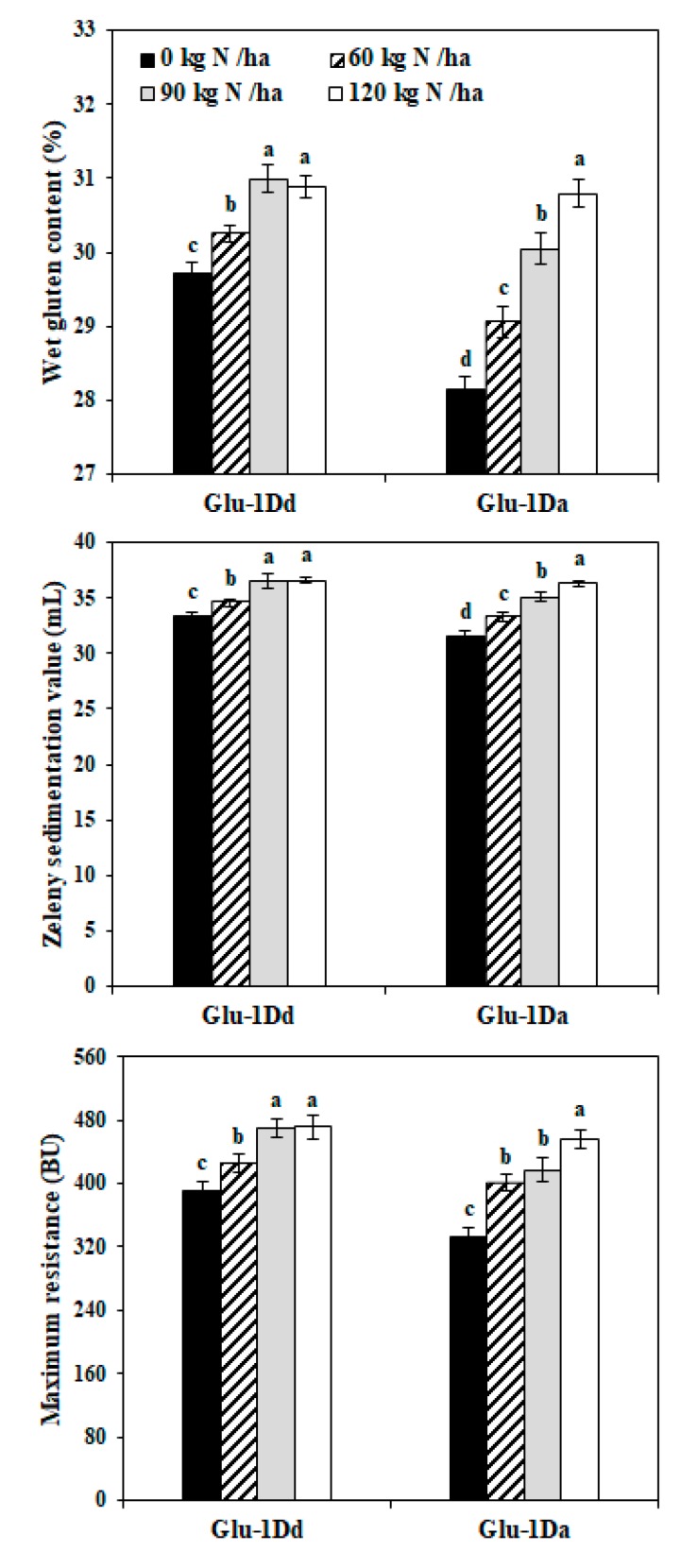
Grain quality traits in the wheat lines with *Glu-1Dd* and *Glu-1Da* under the application of N fertilizer. Same letter in the different columns for each material indicate no significant difference (*p* > 0.05). Data are presented as the mean of two years.

Figure 1 shows the grain traits for the lines with *Glu-1Dd* and *Glu-1Da*, which were evaluated by NIR analysis. With the application of N from 0 to 90 kg N ha^−1^, the wet gluten content, Zeleny sedimentation value, and maximum resistance clearly increased for *Glu-1Dd* and *Glu-1Da*. When N fertilizer was applied at 120 kg N ha^−1^, the values did not differ significantly for *Glu-1Dd*, but they still increased for *Glu-1Da*. Compared with the lines at the 0 kg N ha^−1^, the wet gluten content, Zeleny sedimentation value, and maximum resistance increased by 4.35%, 9.39%, and 20.82% for *Glu-1Dd* at N rate of 90 kg N ha^−1^, respectively, by 9.36%, 15.06%, and 37.26% for *Glu-1Da* at N rate of 120 kg N ha^−1^.

The wet gluten content and Zeleny sedimentation value are well-known indicators for measuring the quality and quantity of wheat gluten, and they are correlated with the end-use quality of dough. The maximum resistance is closely correlated with the viscoelasticity of wheat dough [20]. Wheat with Dx5 + Dy10 has greater grain quality compared with wheat containing the Dx2 + Dy12 subunits [26]. In the present study, the wet gluten content, Zeleny sedimentation value, and maximum resistance were higher for grains with *Glu-1Dd* than those with *Glu-1Da* before applying N fertilizer, which indicates that the wheat with *Glu-1Dd* had better strength of gluten. The component of HMW-GS can affect the grain quality and wheat dough strength [20,25]. N increases the intensity of HMW-GS expression and content of glutenin macropolymer in wheat grains further to improve the grain quality [10]. Thus, the improvement of grain traits was probably attributed to the increment of gluten proteins after the application of N fertilizer. Furthermore, N fertilizer had less effect on the grain quality traits in wheat with *Glu-1Dd* (Dx5 + Dy10) than *Glu-1Da* (Dx2 + Dy12) (Figure 1). This finding is consistent with those obtained by Luo et al. who suggested that genotypes with high stability have weak interactions with the environment [27]. Moreover, when N fertilizer was applied to 120 kg N ha^−1^, the grain yield and NUE–grain yield increased significantly under N application (Supplementary Figure S2). Additionally, the effect of N on the two traits was greater for *Glu-1Dd* compared with *Glu-1Da*. Therefore, the substantial increases in grain biomass maybe cause the small changes in the quality traits of grain with *Glu-1Dd*.

### 3.4. Dynamic Changes in Total Protein and Its Composition Contents of Grains during Grain Development under N Fertilizer Application

**Figure 2:**
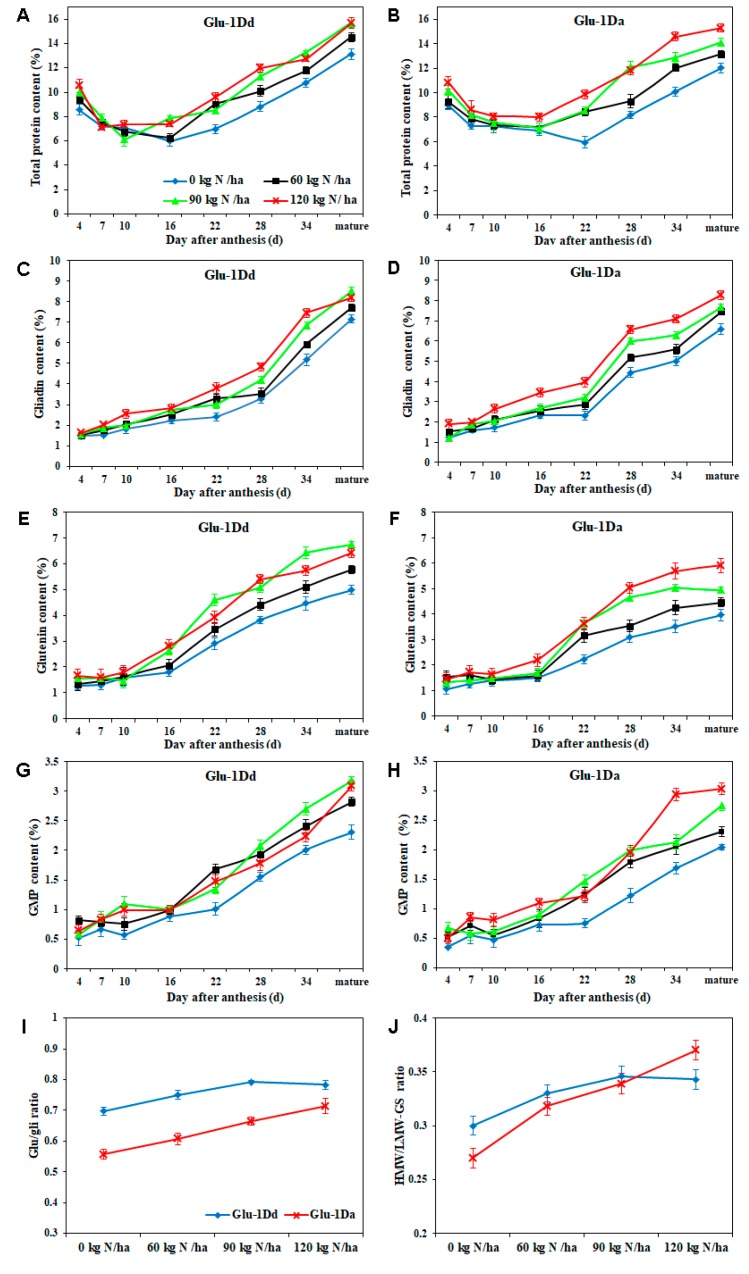
Dynamic accumulation of total protein and its compositions during grain development (**A**‒**H**) and maturity (**I**,**J**) for wheat lines with *Glu-1Dd* and *Glu-1Da* under the application of N fertilizer. (**A**,**B**), total protein content; (**C**,**D**), gliadin content; (**E**,**F**), glutenin content; (**G**,**H**), glutenin macropolymer (GMP) content; (**I**), Glu/gli ratio (ratio of glutenin to gliadin content); (**J**), HMW/LMW-GS ratio (Ratio of HMW-GS to LMW-GS content). Data are presented as the mean of two years. Data of significant difference were analyzed statistically at a level of 0.05.

As shown in Figure 2, the lowest total protein content occurred at 16 DAA for *Glu-1Dd* and 22 DAA for *Glu-1Da* under non-N treatment (Figure 2A,B). However, the lowest total protein content occurred between 7–16 DAA for *Glu-1Dd* and 10–22 DAA for *Glu-1Da* under N application, which suggests that N application could alter the grain protein accumulation pattern. In the early grain development stage, compared with untreated lines, there was no significant difference in the gliadin, glutenin, and GMP contents, however, the contents exhibited a significant increase after 16 DAA until the mature stage (Figure 2C–H), except for the wheat with *Glu-1Dd* at N rate of 120 kg N/ha. Moreover, at maturity, the glu/gli and HMW/LMW-GS ratio increased significantly with the N rate from 0 to 120 kg N ha^−1^ for *Glu-1Da*, whereas for *Glu-1Dd*, the ratios increased markedly to maximum at N rate of 90 kg N ha^−1^, but decreased slightly at N rate of 120 kg N ha^−1^ (Figure 2I,J). Compared with the untreated lines of the two NILs at the maturity, the total protein, gliadin, glutenin, GMP, glu/gli ratio, and HMW/LMW-GS ratio increased by 19.68%, 19.42%, 35.35%, 37.76%, 13.56%, and 15.33% for *Glu-1Dd* at N rate of 90 kg N ha^−1^, respectively, increased by 27.14%, 25.73%, 49.37%, 28.14%, 49.17%, and 37.04% for *Glu-1Da* at N rate of 120 kg N ha^−1^, respectively.

The total protein and its composition contents play important roles in determining the end-use quality of wheat dough [12,26,28]. The application of N accelerated and increased the accumulation of total protein, gliadin, glutenin, and GMP during grain-filling, thereby resulting in higher contents at maturity. These results are consistent with previous studies that the application of N in the booting stage increased the total protein, gliadin, and glutenin contents, as well as the GMP, glu/gli ratio, HMW/LMW-GS ratio [4,6]. The effect of N fertilizer on the total protein and its compositions in *Glu-D1a* was greater than that in *Glu-D1d*, indicating that N favored the accumulation of gliadin, glutenin, and GMP in wheat with weaker gluten (low GPC), which does not agree with a previous report [10], but it is consistent with a study [15]. Compared with other topdressing stages, the gliadin and glutenin contents of the mature grains were highest under the same N application in the booting stage, as well as the GMP, glu/gli ratio, and HMW/LMW-GS ratio, thereby obtaining a better quality of gluten [4]. Therefore, in order to improve the quality of wheat, it is necessary to study the effect of N application on the dynamic accumulation of gliadin, glutenin, and GMP during grain development in the booting stage.

### 3.5. Changes in Sulfhydryl Groups (-SH) and Disulfide Bonds (-S-S-) in Gluten with Variations in HMW-GS at the Glu-D1 Locus under Different N Treatments

**Figure 3:**
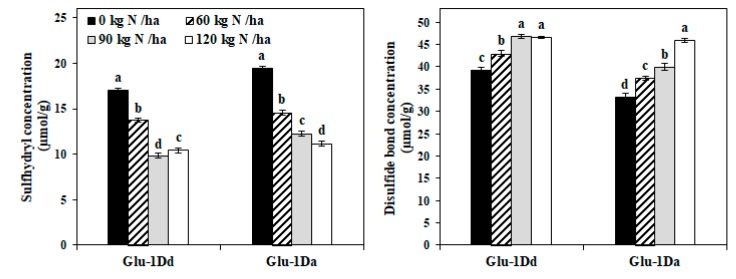
Concentration of sulfhydryl groups and disulfide bonds in lines with *Glu-1Dd* and *Glu-1Da* under N treatment. Same letter in the different columns for each material indicate no significant difference (*p* > 0.05). Data are presented as the mean of two years.

The -SH and -S-S- were usually quantified to confirm the formation of covalent bonds and cross-linking of the proteins [29]. The effects of N treatments on the -SH and -S-S- were showed in Figure 3. The concentration of -SH in *Glu-1Dd* and *Glu-1Da* gluten decreased with the application of N fertilizer, except for a slight increase in *Glu-1Dd* at rate of 120 kg N ha^−1^, whereas the -S-S- concentration exhibited the opposite trend. Compared with the untreated lines, the -SH concentration decreased by 39.49% and 42.54% for *Glu-1Dd* at N rate of 90 kg N ha^−1^ and for *Glu-1Da* at 120 kg N ha^−1^, respectively, while the -S-S- concentrations increased by 19.57% and 38.12%, respectively, for *Glu-1Dd* at N rate of 90 kg N ha^−1^ and for *Glu-1Da* at 120 kg N ha^−1^.

Disulfide bonds play important roles in determining the structural properties of gluten proteins [28]. The cysteine residues existed in HMW-GS and LMW-GS are involved in inter- and intra-molecular disulfide bonding in the formation of GMPs and play an important role in the function of HMW-GSs [2,29]. Our results show that the native gluten with *Glu-D1d* exhibited lower the -SH and higher -S-S- concentration compared with gluten with *Glu-1Da*, which was probably attributed to the additional cysteine number in Dx5 [28] that formed more -S-S- bonds between HMW-GSs to lead in greater polymerization of glutenin. Under the application of N fertilizer, the changes in -SH and -S-S- concentration were greater in *Glu-D1a* gluten than *Glu-D1d*, which suggested that the N fertilizer promoted quick conversion from -SH to S-S bonds, but especially in gluten with *Glu-1Da* during the formation of gluten proteins. Zhao et al. [30] found that the de-polymerization and re-polymerization of the high molecular weight fraction of gluten are mainly caused by the interchanges between S-S bonds and -SH groups, which agrees with the results of GMP and S-S bonds in the present study. Recent studies suggested that the application of N fertilizer up-regulated the expression of protein disulfide isomerase [31], peptidyl-prolyl cis–trans isomerase and small ubiquitin-related modifier 1 [32], thereby promoting the polymerization of glutenin. Thus, changes in the S-S bonds of gluten containing *Glu-1Dd* and *Glu-1Da* were related to the expression of genes involving into protein polymerization during the formation of gluten proteins.

### 3.6. Effect of N Application on the Secondary Structures of Gluten with Variations in HMW-GS at the Glu-D1 Locus

**Figure 4:**
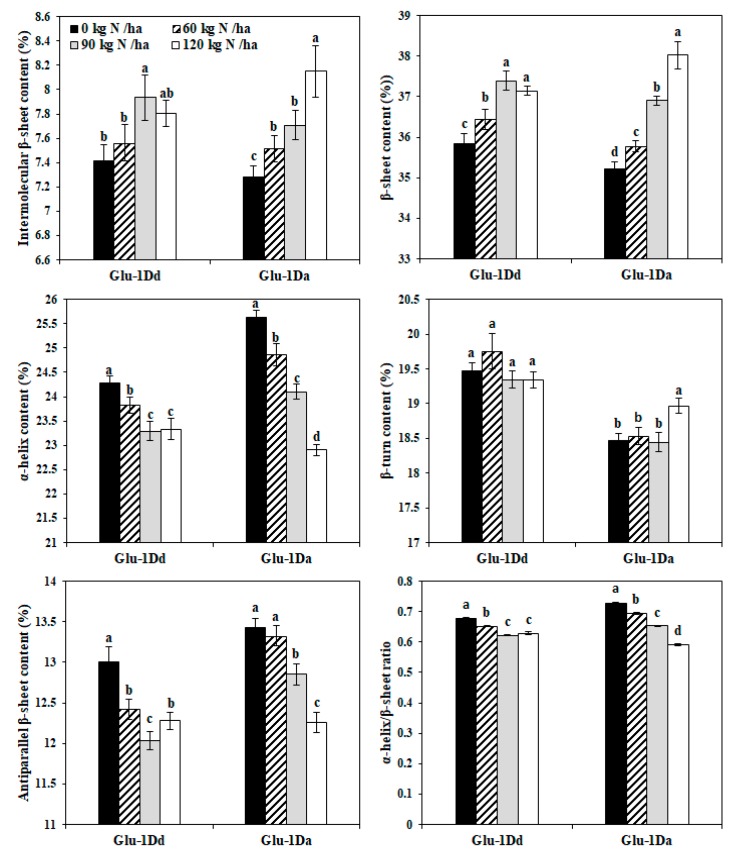
Percentages of secondary structure of gluten containing *Glu-1Dd* and *Glu-1Da* under N treatment. Same letter in the different columns for each material indicate no significant difference (*p* > 0.05). Data are presented as the mean of two years.

The effects of N on the secondary structures of dry gluten with *Glu-1Dd* and *Glu-1Da* are shown in Figure 4. The intermolecular β-sheet and β-sheet contents increased for *Glu-1Dd* under N application at rates from 0 to 90 kg N ha^−1^, and they then decreased slightly at 120 kg N ha^−1^, while the contents increased significantly for *Glu-1Da* under N fertilizer application from 0 to 120 kg N ha^−1^. The responses to N of the antiparallel β-sheets, α-helices, and α-helix to β-sheet ratios were opposite to those of the β-sheet contents. The responses of β-turn contents were irregular for gluten with *Glu-1Dd* and *Glu-1Da* under the different N treatments. Compared with the untreated lines, the intermolecular β-sheet and β-sheet increased by 7.08%, and 4.34% for *Glu-1Dd* at N rate of 90 kg N ha^−1^, respectively, and by 11.95% and 8.01% for *Glu-1Da* at 120 kg N ha^−1^, respectively, while the antiparallel β-sheets, α-helices, and α-helix to β-sheet ratios decreased by 7.42%, 4.06%, and 8.05% for *Glu-1Dd* at 90 kg N ha^−1^, respectively, and by 8.72%, 10.62%, and 17.24% for *Glu-1Da* at 120 kg N ha^−1^, respectively.

A higher α-helix content indicates a weaker protein network and it is negatively correlated with the viscoelasticity of dough, whereas a higher β-sheet content indicates a stronger protein network and it is positively correlated with the dough viscoelasticity [17,23]. In the present study, the changes in the secondary structure of gluten exhibited different patterns in response to the different N application between cultivars. The β-sheet content of gluten with *Glu-1Dd* was highest at 90 kg N ha^−1^, whereas that with *Glu-1Da* was highest at 120 kg N ha^−1^, thus both of the two were expected to obtain stronger gluten network and dough viscoelasticity. Rearrangements of the protein spatial conformation are caused by interactions between covalent bonds (-S-S-) and non-covalent bonds [33], thus the changes in conformations of gluten secondary structure were likely to attribute to changes in the S-S bonds after application of N fertilizer. Moreover, the N application had stronger effects on the conformations of gluten with *Glu-1Da* gluten than *Glu-1Dd*, which agrees with previous findings that wheat with superior alleles (e.g., Dx5 + Dy10) is less affected by the growing conditions [26].

### 3.7. Effects of N on the Thermal Properties of Gluten with Variations in HMW-GS at the Glu-D1 Locus

**Figure 5:**
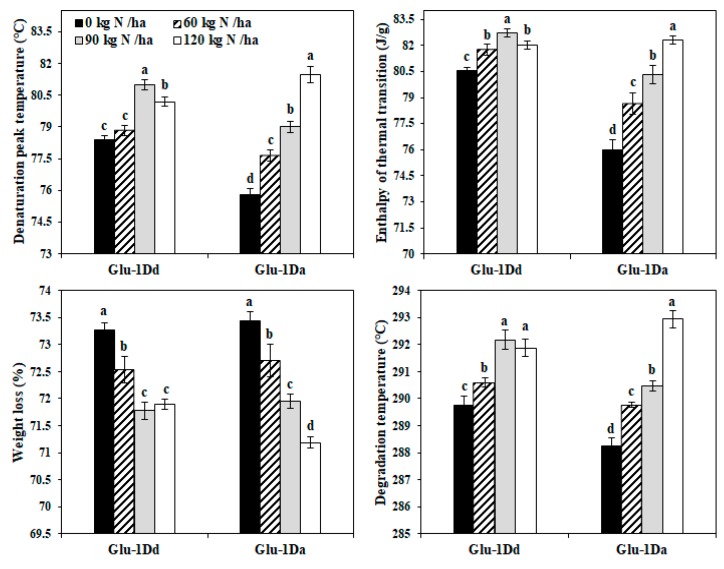
Thermal properties obtained from the gluten containing *Glu-1Dd* and *Glu-1Da* under different application N rates. The same letter in the different columns for each material indicates no significant difference (*p* > 0.05). Data are presented as the mean of two years.

The thermal analyses of gluten with *Glu-1Dd* and *Glu-1Da* obtained under the application of N fertilizer are showed in Figure 5. The denaturation peak temperature (T_p_), enthalpy of thermal transition (ΔH), and degradation temperature (T_d_) increased significantly for *Glu-1Dd* and *Glu-1Da* with N rates from 0 to 120 kg N ha^−1^, except for a slight decrease for *Glu-1Dd* at 120 kg N ha^−1^. However, for *Glu-1Dd*, the weight loss decreased with N rates from 0 to 90 kg N ha^−1^, but there was no significant change when N was applied to 120 kg N ha^−1^, whereas the weight loss decreased significantly at all rates from 0 to 120 kg N ha^−1^ for *Glu-1Da*. Compared with the untreated lines, the T_p_ and ΔH increased by 3.28% and 2.71% for *Glu-1Dd* at 90 kg N ha^−1^, respectively, by 7.47% and 8.29% for *Glu-1Da* at 120 kg N ha^−1^, respectively.

A higher T_p_ is associated with a more cross-linked gluten network [18], and a higher ΔH indicates that the gluten protein forms a more regular network structure [34]. The T_p_ and ΔH values were highest at 90 kg N ha^−1^ for *Glu-1Dd* and 120 kg N ha^−1^ for *Glu-1Da* in our experiment, which indicates that the both exhibited high temperature resistance due to their stronger gluten network structures. The increased T_d_ and reduced weight loss indicate the formation of a more compact and stronger gluten network in the dough [18]. For *Glu-1Da*, the T_d_ and weight loss were highest and lowest at 120 kg N ha^−1^, respectively, whereas for Glu-1Dd, the T_d_ was highest and weight loss was lowest at 90 kg N ha^−1^ (Figure 5). The S-S bond is an important functional bond in the tertiary structure of proteins and it is closely related to the structural stability [34,35]. Our result shows that the increment in the -S-S- concentrations and β-sheet proportion in gluten containing *Glu-1Da* was greater than those in *Glu-1Dd* after applying N fertilizer, probably accounting for the higher thermal stability of gluten containing *Glu-1Da* compared to *Glu-1Dd*. Therefore, we speculated that the moderate amount of N in the booting stage improved the thermal properties of gluten by affecting the network structure, especially in the wheat with weaker gluten (*Glu-1Da*).

### 3.8. Dough Mechanical Mixing Properties of NILs

**Figure 6:**
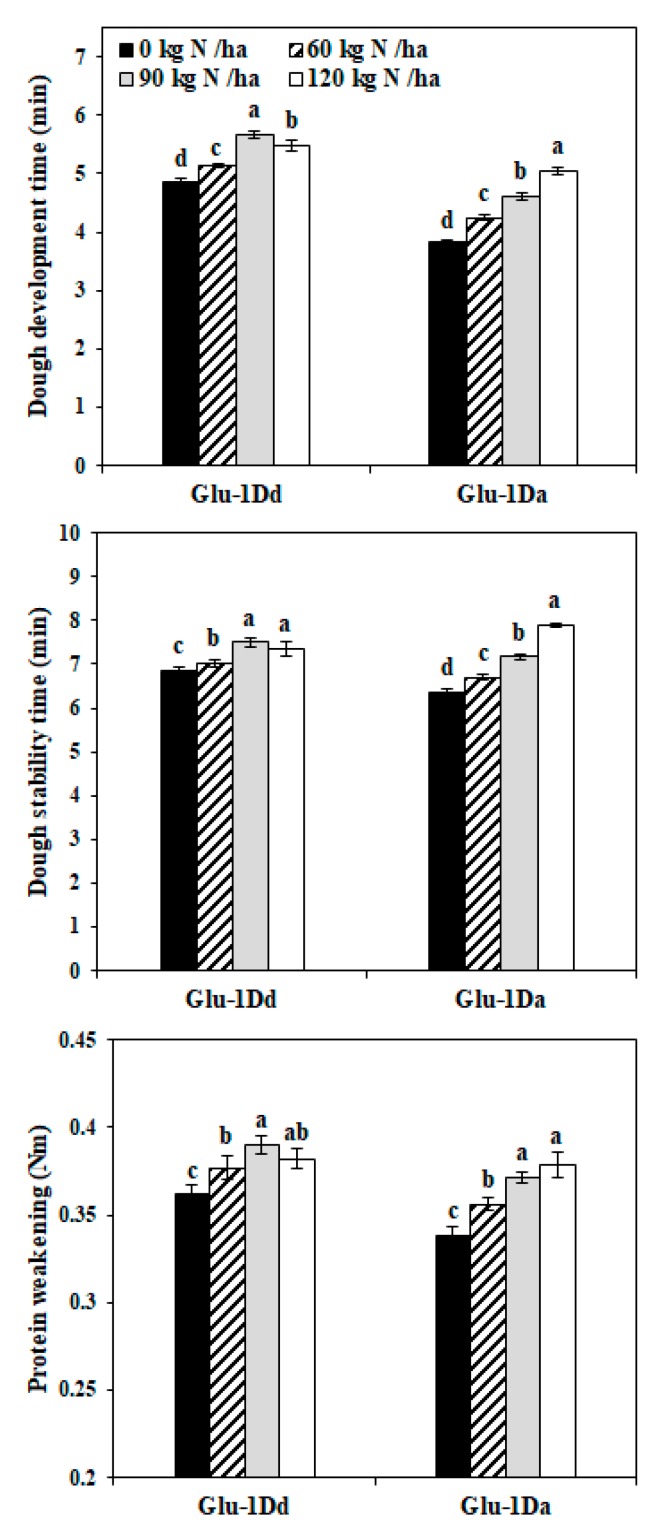
Mixing properties of dough in lines with *Glu-1Dd* and *Glu-1Da* under N treatment. The same letter in the different columns for each material indicates no significant difference (*p* > 0.05). Data are presented as the mean of two years.

The dough mixing properties reflecting gluten strength was measured using a Mixolab system (Figure 6). For *Glu-1Dd*, the dough development time, dough stabilization time, and protein weakening exhibited increasing trends as the application of N increased from 0 to 90 kg N ha^−1^, whereas the values decreased under the application of 120 kg N ha^−1^. However, the values determined for *Glu-1Da* increased significantly as the application of N increased from 0 to 120 kg N ha^−1^. Compared with the untreated lines, the dough development time, dough stability time, and protein weakening increased by 16.77%, 9.12%, and 7.88% for *Glu-1Dd* at 90 kg N ha^−1^, respectively, by 31.71%, 24.25%, and 11.82% for *Glu-1Da* at 120 kg N ha^−1^, respectively.

The dough development time and dough stability time were used as indicators of the gluten strength [36], and the protein weakening was positively correlated with the SDS sedimentation volume, dough extensibility, and loaf volume [24]. The late application of N fertilizer increased the SDS sedimentation value and mixograph parameters [27]. Therefore, the *Glu-1Dd* at 90 kg N ha^−1^ and *Glu-1Da* at 120 kg N ha^−1^ could obtain a better gluten strength and elasticity. Moreover, the changes induced by N fertilizer were greater in the dough development time, dough stability time, and protein weakening for *Glu-1Da* than those for *Glu-1Dd*. The gluten strength in the wheat with Dx2 + Dy12 was weaker compared with that containing Dx5 + Dy10 [26], so the *Glu-1Da* (Dx2 + Dy12) was probably more sensitive to the environment, thereby leading to greater changes after the application of N. These results suggest that applying a moderate amount of N fertilizer in the booting stage could improve the dough mixing properties, which is not consistent with a previous report that late N application had no influence on the baking quality of wheat [6]. This difference may be related to variations in the available N in the soil and the diverse genotypes.

### 3.9. Effects of N on the Microstructure of Gluten with Variations in HMW-GS at the Glu-D1 Locus

**Figure 7:**
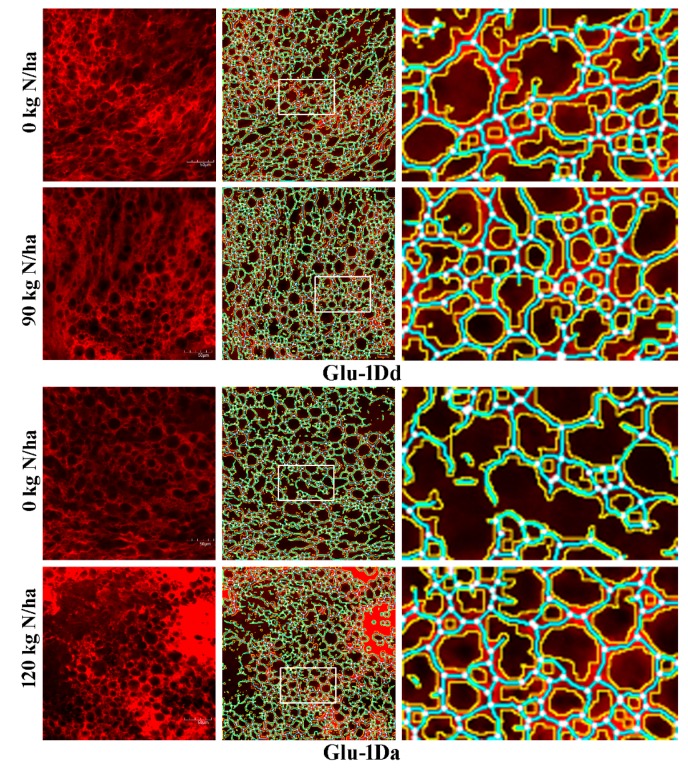
Protein network analysis for dough produced with *Glu-1Dd* and *Glu-1Da* under N application. The images in the left column are the original CLSM images, and the scale bar represents 50 µm. The images in the middle column were processed with AngioTool. The images in the right column show the enlarged areas selected from the processed images highlighted by the white boxes, where the junctions are shown in white, the protein skeleton in blue, and the protein outline/area in yellow.

The GMP, gluten thermal and structural characteristics, dough mixing properties were improved to a greater extent at N rate of 90 kg N ha^−1^ for *Glu-1Dd* and 120 kg N ha^−1^ for *Glu-1Da*, so we quantitatively analyzed the gluten microstructures using CLSM. Figure 7 showed that compared with non–N fertilizer application, the microstructures seem denser and more compact in the dough obtained with *Glu-1Da* and *Glu-1Dd* at 90 kg N ha^−1^ and 120 kg N ha^−1^, respectively, indicating that N application increased the protein contents and -S-S- concentrations, which agrees with the results of Figure 2 and Figure 3. The CLSM images were analyzed further to quantify the gluten network structure. As shown in Table 1, applying N fertilizer significantly increased the protein area, number of junctions, junction density, total protein length, branching rate, and lacunarity of the networks of glutens containing *Glu-1Da* and *Glu-1Dd*. However, the protein endpoints and endpoint rates were decreased dramatically by the application of N fertilizer.

Higher protein area (indicating a low aperture area) and protein junction (related to the junction density and branching rate) values indicate that the gluten network has greater connectivity [37]. The lacunarity represents the amount of gaps and irregularities in a protein network and it is positively correlated with dough development time and dough stability time, whereas the protein endpoints and endpoint rate are negatively correlated with those properties [25]. The quantitative results obtained by CLSM indicated that the application of N made the wheat gluten microstructure more even and denser in the two NILs (Table 1 and Figure 7), thereby obtaining stronger gluten networks. Moreover, applying N had a greater effect on the microstructure of gluten containing *Glu-1Da* than *Glu-1Dd*, which agrees with the results obtained by near-infrared reflectance, glutenin polymerization, secondary structure, and thermal property analyses. Therefore, the moderate amount of N application in the booting stage improved the dough strength by affecting the structure of gluten, but especially with inferior glutenin subunits.

### 3.10. Correlation Analysis of the Quality–related Parameters

As shown in Supplementary Table S1, the GMP was closely correlated with gluten secondary structure, dough mixing properties, and the parameters of protein network analysis, which was consistent with the result of previous studies [20,25]. The thermal stability and disulfide bond concentration of gluten also was found to relate with the GMP, gluten secondary structures, dough mixing properties, and protein network analysis. Therefore, this study may provide an insight into the effect of N fertilizer in the booting stage on the quality formation of wheat.

## 4. Conclusions

In this study, we investigated the effects of N fertilizer application in two wheat NILs (*Glu-1Da* and *Glu-1Dd*) on the polymerization of glutenin during grain development as well as the structural–thermal properties of gluten in the booting stage. The application of N accelerated and increased the accumulation of total protein, glutenin, glu/gli, HMW/LMW-GS, and GMP in the grain, increased the disulfide bond concentration, improved the gluten secondary and micro structure, and increased greater thermal stability and dough mixing properties. In addition, the response to N fertilizer was greater in wheat with the *Glu-1Da* allele than *Glu-1Dd*. Moreover, the correlation analysis of the quality-related parameters provides strong evidence once again that GMP can be used as an indicator associated with the quality of wheat. Therefore, applying N in the booting stage was effective at improving the gluten structural and thermal properties of wheat, especially with inferior glutenin subunits.

## Figures and Tables

**Table 1:** Parameters determined by AngioTool to reflect the protein networks in the gluten containing *Glu-1Dd* and *Glu-1Da* dough.

Line	N Rate (kg N /ha)	Protein Area (×10^4^ μm^2^)	Protein Junctions (×10^2^)	Junction Density (×10^−3^)	Total Protein Length (×10^3^ µm)	Protein Endpoints (× 10^2^)	Lacunarity (×10^−2^)	Branching Rate (×10^−3^)	Endpoint Rate (×10^−3^)
*Glu-1Dd*	0	12.48 ± 0.22^b^	11.88 ± 0.12^b^	4.55 ± 0.07^b^	20.62 ± 0.27^b^	5.55 ± 0.03^a^	6.18 ± 0.06^b^	9.52 ± 0.21^b^	4.45 ± 0.08^a^
	90	12.91 ± 0.15^a^	13.20 ± 0.23^a^	5.06 ± 0.12^a^	22.29 ± 0.13^a^	4.88 ± 0.10^b^	6.75 ± 0.14^a^	10.22 ± 0.14^a^	3.78 ± 0.06^b^
*Glu-1Da*	0	11.06 ± 0.11^b^	10.77 ± 0.21^b^	4.13 ± 0.17^b^	19.42 ± 0.20^b^	5.33 ± 0.09^a^	5.76 ± 0.11^b^	9.73 ± 0.09^b^	4.81 ± 0.03^a^
	120	12.64 ± 0.20^a^	14.00 ± 0.42^a^	5.37 ± 0.28^a^	21.14 ± 0.95^a^	4.69 ± 0.05^b^	6.55 ± 0.12^a^	11.07 ± 0.25^a^	3.72 ± 0.08^b^

Values represent mean ± SD (*n* = 10). Different letters in the same column for each material are significantly different (*p* < 0.05).

## Supplementary Materials

The following are available online at https://www.mdpi.com/2304-8158/9/3/353/s1, Figure S1: Separation and identification of gliadins and glutenins. **A,** Gliadins from the two NILs separated by A-PAGE: lane 1, *Glu-1Dd*; lane 2, *Glu-1Da*. **B,** Glutenins separated by SDS-PAGE from the two NILs and five wheat varieties as controls: lane 1, *Glu-1Dd*; lane 2, *Glu-1Da*; lane 3, Pompei (null, Bx6 + By8, Dx2 + Dy12); lane 4, Jin47 (null, Bx7 + By9, Dx2 + Dy12); lane 5, Guadalupe (null, Bx13 + By19, Dx5 + Dy10); lane 6, Lankao Teaizao (Ax1, Bx7 + By8, Dx2 + Dy12). **C**, Separation of the glutenins from the two NILs differs at *Glu-D1* locus by RP-HPLC. Figure S2: Plant nitrogen (N) uptake, recovery efficiency, nitrogen use efficiency (NUE)–total protein yield, NUE–GMP yield, grain yield, and NUE–grain yield of the wheat lines with *Glu-1Dd* and *Glu-1Da* under different N management strategies. Same letter in the different columns for each material indicate no significant difference (*p* > 0.05). Data are present as mean of two years. Table S1: Pearson correlation coefficients between GMP, different secondary structures, thermal stability, disulfide bond concentrations, dough mixing properties, and the parameters of protein network analysis.
