# Supplementary material for: Effects of Nitrogen Application in the Wheat Booting Stage on Glutenin Polymerization and Structural–Thermal Properties of Gluten with Variations in HMW-GS at the Glu-D1 Locus
Source: Foods. 2020 Mar 18;9(3):353. doi: 10.3390/foods9030353 (PMC7143320; doi:10.3390/foods9030353)
Supplement: Supplementary file 1 [file foods-09-00353-s001.pdf]

Article

Effects of Nitrogen Application in the Wheat Booting Stage on Glutenin Polymerization and Structural–Thermal Properties of Gluten with Variations in HMW-GS at the *Glu-D1* Locus

Lijun Song †, Liqun Li †, Liye Zhao, Zhenzhen Liu, and Xuejun Li *

State Key Laboratory of Crop Stress Biology in Arid Areas and College of Agronomy, Northwest A&F University, Yangling, Shaanxi 712100, China; lijunsongNWAFU@163.com (L.S.); liliqun@nwsuaf.edu.cn (L.L.); liyezhao5526@163.com (L.Z.); zhenzhenliu1717@163.com (Z.L.)

* Correspondence: xuejun@nwsuaf.edu.cn (X.L.); Tel./Fax: +86-29-8708-2022

† These authors contributed equally to this work.

Supplementary data

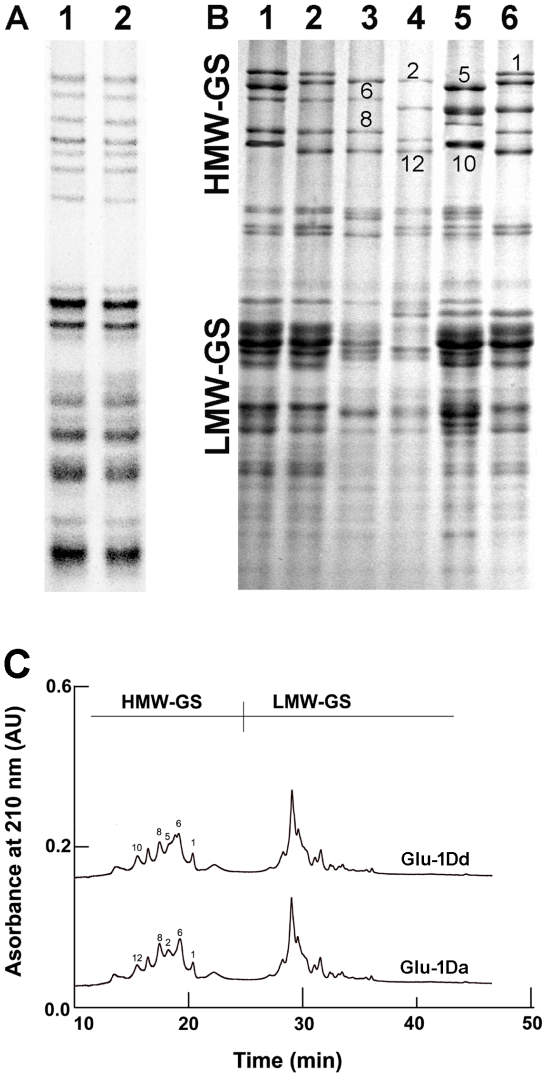

Figure S1. Separation and identification of gliadins and glutenins. **A**, Gliadins from the two NILs separated by A-PAGE: lane 1, *Glu-1Dd*; lane 2, *Glu-1Da*. **B**, Glutenins separated by SDS-PAGE from the two near-isogenic lines (NILs)and five wheat varieties as controls: lane 1, *Glu-1Dd*; lane 2, *Glu-1Da*; lane 3, Pompei (null, Bx6+By8, Dx2+Dy12); lane 4, Jin47 (null, Bx7+By9, Dx2+Dy12); lane 5, Guadalupe (null, Bx13+By19, Dx5+Dy10); lane 6, Lankao Teaizao (Ax1, Bx7+By8, Dx2+Dy12). **C**, Separation of the glutenins from the two NILs differs at *Glu-D1* locus by RP-HPLC.

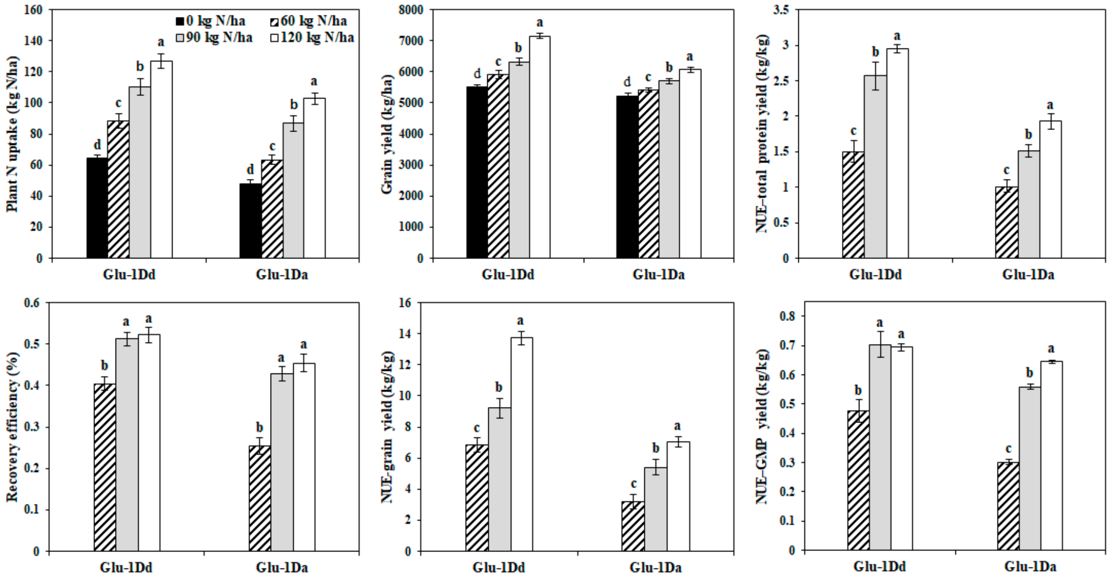

Figure S2. Plant nitrogen (N) uptake, recovery efficiency, nitrogen use efficiency (NUE) –total protein yield, NUE–GMP yield, grain yield, and NUE–grain yield of the wheat lines with Glu-1Dd and Glu-1Da under different N management strategies. Same letter in the different columns for each material indicate no significant difference ($p > 0.05$). Data are present as mean of two years.

Table S1 Pearson correlation coefficients between GMP, different secondary structures, thermal stability, disulfide bond concentrations, dough mixing properties, and the parameters of protein network analysis.

	GMP	β-sheets	Intermolecular β-sheets	α-helices	α-helix/β-sheet ratio	Denaturation peak temperature	Enthalpy of thermal transition	Weight loss	Degradation temperature	Disulfide bonds	Dough development time	Dough stability time	Protein weakening	Lacunarity	Branch rate
β-sheets	0.998**	1													
Intermolecular β-sheets	0.960**	0.966**	1												
α-helices	-0.966**	-0.970**	-0.963**	1											
α-helix/β-sheet ratio	-0.991**	-0.992**	-0.965**	0.989**	1										
Denaturation peak temperature	0.926**	0.912**	0.891**	-0.868**	-0.901**	1									
Enthalpy of thermal transition	0.905**	0.881*	0.802*	-0.875**	-0.903**	0.891**	1								
Weight loss	-0.937**	-0.953**	-0.960**	0.739	0.950**	-0.782*	-0.740	1							
Degradation temperature	0.893**	0.899**	0.884*	-0.888**	-0.882**	0.765	0.749	-0.853**	1						
Disulfide bonds	0.893**	0.897**	0.915*	-0.884**	-0.911**	0.870**	0.808*	-0.896**	0.680	1					
Dough development time	0.967**	0.964**	0.904**	-0.962**	-0.971**	0.853**	0.920*	-0.891**	0.927**	0.814*	1				
Dough stability time	0.721*	0.727*	0.828*	-0.781*	-0.759*	0.809*	0.639	-0.718*	0.543	0.878**	0.625	1			
Protein weakening	0.883**	0.874**	0.874**	-0.869**	-0.899**	0.803*	0.877**	-0.863**	0.720*	0.930**	0.857**	0.725*	1		
Lacunarity	0.972*	0.962*	0.882	-0.894	-0.932	0.933	0.974*	-0.793	0.904	0.757	0.962*	0.611	0.947*	1	
Branch rate	0.963*	0.966*	0.984*	-0.985*	-0.983*	0.913	0.866	-0.921*	0.769	0.970*	0.957**	0.878*	0.919**	0.892	1
End-point rate	-0.955*	-0.948*	-0.925	0.933	0.949	-0.983*	-0.950*	0.816	-0.745	-0.909	0.850	0.848*	0804	-0.935	-0.972

* And ** indicate significant correlations at $p < 0.05$ and $p < 0.01$, respectively.
